# In vivo catecholaminergic metabolism in the medial prefrontal cortex of ENU2 mice: an investigation of the cortical dopamine deficit in phenylketonuria

**DOI:** 10.1007/s10545-012-9473-2

**Published:** 2012-03-24

**Authors:** Tiziana Pascucci, Giacomo Giacovazzo, Diego Andolina, David Conversi, Fabio Cruciani, Simona Cabib, Stefano Puglisi-Allegra

**Affiliations:** 1 Department of Psychology and Centre “Daniel Bovet”, “Sapienza” University, via dei Marsi 78, 00185 Rome, Italy; 2 Santa Lucia Foundation, European Centre for Brain Research CERC, Rome, Italy

## Abstract

**Objective:**

Phenylketonuria (PKU) is an inherited metabolic disease characterized by plasma hyperphenylalaninemia and several neurological symptoms that can be controlled by rigorous dietetic treatment. The cellular mechanisms underlying impaired brain functions are still unclear. It has been proposed, however, that phenylalanine interference in cognitive functions depends on impaired dopamine (DA) transmission in the prefrontal cortical area due to reduced availability of the precursor tyrosine. Here, using Pah^enu2^ (ENU2) mice, the genetic murine model of PKU, we investigated all metabolic steps of catecholamine neurotransmission within the medial preFrontal Cortex (mpFC), availability of the precursor tyrosine, synthesis and release, to find an easy way to reinstate normal cortical DA neurotransmission.

**Methods and results:**

Analysis of blood and brain levels of tyrosine showed reduced plasma and cerebral levels of tyrosine in ENU2 mice. Western blot analysis demonstrated deficient tyrosine hydroxylase (TH) protein levels in mpFC of ENU2 mice. Cortical TH activity, determined *in vivo* by measuring the accumulation of l-3,4-dihydroxyphenylalanine (L-DOPA) in mpFC after inhibition of L-aromatic acid decarboxylase with NSD-1015, was reduced in ENU2 mice. Finally, a very low dose of L-DOPA, which bypasses the phenylalanine-inhibited metabolic steps, restored DA prefrontal transmission to levels found in healthy mice.

**Conclusion:**

The data suggests that a strategy of using tyrosine supplementation to treat PKU is unlikely to be effective, whereas small dose L-DOPA administration is likely to have a positive therapeutic effect.

## Introduction

Phenylketonuria (PKU; McKusick 2610600) is an inherited metabolic disease caused by a deficiency of the enzyme phenylalanine hydroxylase, which is necessary to convert phenylalanine to tyrosine. This results in accumulation of phenylalanine (> 20 mg/dl), known as hyperphenylalaninemia, and reduction of tyrosine concentrations in the blood and brain. Treatment of PKU requires maintaining blood phenylalanine within an acceptable range (between 2 and 10 mg/dl) by restricting phenylalanine from the diet. If the disease is untreated, patients develop severe mental retardation and neuropathological signs. Compliance with a rigid low phenylalanine diet is difficult (Giovannini et al 2007; MacDonald 2000), and it is still unknown when or if the diet can be safely interrupted (De Roche and Welsh 2008; Stemerdink et al 2000; Diamond et al 1994). Indeed, evidence indicates that even mildly elevated blood phenylalanine levels induce deficits in cognitive functions involving the prefrontal cortical area (Brumm et al 2004: Channon et al 2004; De Roche and Welsh 2008; Diamond et al 1994; Huijbregts et al 2002; Leuzzi et al 2004; Schmidt et al 1994; Smith et al 2000; White et al 2002; Stemerdink et al 2000), in particular executive abilities. This suggests that excess phenylalanine interferes with cortical functioning. The medial prefrontal cortex (mpFC) is widely innervated by biogenic aminergic neurons, which have a major role in emotion and cognitive functions (Arnsten and Robbins 2002; Lapiz and Morilak 2006; Aston-Jones and Cohen 2005; Goldman-Rakic 1999; Clarke et al 2004, 2005, 2006; Walker et al 2009). Moreover, there are reports of reduced levels of biogenic amines in *post mortem* brain tissue (McKean 1972), low levels of biogenic amine metabolites in cerebrospinal fluid of patients with hyperphenylalaninemia (Bonafé et al 2001; Butler et al 1981) and reduced brain amine levels and metabolism in PAH^enu2^ mice (ENU2) (Puglisi-Allegra et al 2000; Pascucci et al 2002, 2008), that is, the genetic murine model of PKU. Dopamine (DA) is the most studied among cerebral biogenic amines (Diamond 2007; Joseph and Dyer 2003). Although reduction of cerebral DA metabolism has been reported in PKU patients (Diamond et al 1994; Hanley et al 2000; Krause et al 1985; Guttler and Lou 1986; Lou et al 1987; Luciana et al 2004; Lykkelund et al 1988; McKean and Peterson 1970; Paans et al 1996) and ENU2 mice (Joseph and Dyer 2003; Pascucci et al 2009; Puglisi-Allegra et al 2000; Smith and Kang 2000), DA metabolism in mpFC of PKU organisms has not been investigated until now. Therefore, it is difficult to determine the mechanisms by which high blood phenylalanine levels reduce cortical DA biosynthesis (De Groot et al 2010; Martynyuk et al 2010).

ENU2 mice represent a qualified model for clarifying neurochemical deficits in pFC of PKU organisms, because they are characterized by a biochemical phenotype that closely resembles untreated human PKU, as well as by reduced enzymatic activity of phenylalanine hydroxylase, high blood phenylalanine levels, hypomyelination, biochemical and behavioural deficits (Andolina et al 2010; Cabib et al 2003; Embury et al 2007; Glushakov et al 2005; Joseph and Dyer 2003; Martynyuk et al 2005; Pascucci et al 2002, 2008, 2009; Puglisi-Allegra et al 2000; Smith and Kang 2000; Zagreda et al 1999). In particular, previous data showed deficits in DA and norepinephrine (NE) metabolism in the mpFC of ENU2 mice (Joseph and Dyer 2003; Puglisi-Allegra et al 2000; Pascucci et al 2009). Since DA availability in mpFC is very important in executive functions, the elucidation of the mechanism by which phenylalanine reduces cortical DA metabolism is essential. Thus, the present study was aimed at investigating catecholaminergic metabolism in the mpFC of ENU2 mice and at suggesting strategy to reinstate normal cortical catecholamine levels.

First, we investigated tyrosine blood and brain levels in order to evaluate the influence of excess phenylalanine on brain availability of catecholamine precursor. Second, we evaluated expression and *in vivo* activity of the tyrosine hydroxylase (TH) enzyme in mpFC of ENU2 mice. Finally, we evaluated the effect of l-3,4-dihydroxyphenylalanine (L-DOPA), the direct DA precursor, on activation of the frontal cortical catecholaminergic transmission in the presence of high circulating phenylalanine levels. Since restraint stress is known to induce a clear-cut increase of amine outflow in the mpFC of rodents (Cuadra et al 2001; Matuszewich et al 2002; Pascucci et al 2007), and phenylketonuric mice are unable to activate catecholamine release under stress (Pascucci et al 2009), we assessed the effect of L-DOPA on cortical catecholamine release of restrained ENU2 mice.

## Materials and methods

### Animals

Homozygous (-/-) Pah^Enu2^ (ENU2) and (+/+) Pah^Enu2^ (WT) male mice of the background strain (BTBR) were obtained from heterozygous mating. Genetic characterization was performed on DNA prepared from tail tissue using the Easy DNA Kit (Invitrogen, Carlsbad, CA, USA). The enu2 mutation was detected after PCR amplification of exon 7 of the Pah gene and digestion with Alw261 restriction enzyme (Promega corporation, Madison, Wi, USA) as described (Pascucci et al 2008). At postnatal day 28, animals (sex matched) were housed 2-4 per standard breeding cage with food and water *ad libitum* on a 12:12h dark: light cycle (light on 07.00 am -07.00 pm h). Experiments started when animals reached 8 weeks of age. All mice were housed individually 24 h before surgery for microdialysis. Naive animals were used for each experiment.

All experiments were conducted in accordance with European legislation (EEC no. 86/609), Italian national legislation (DL no. 116/92) governing the use of animals for research, and the guidelines of the National Institutes of Health on the use and care of laboratory animals.

### Drugs

Chloral hydrate, NSD-1015, and L-DOPA were purchased from Sigma-Aldrich (St. Luis, MO, USA). NSD-1015 was dissolved in artificial CSF and perfused through the microdialysis probe. Chloral hydrate (450 mg/kg) and L-DOPA (0.5, 1, 2.5 mg/kg) were dissolved in saline (0.9 % NaCl) and injected i.p. in a volume of 10 ml/kg.

### Brain and blood assay

For brain and blood phenylalanine and tyrosine assay, WT (n = 6) and ENU2 (n = 6) mice were sacrificed by decapitation. Brains and blood were prepared for biochemical analysis.

First, the brains were removed, frozen and stored in liquid nitrogen until the day of biochemical assay. Frozen whole brains were weighed and homogenized in 0.05M HClO4 (1:100 ml/mg). The homogenates were centrifuged at 10000 x g for 20 min at 4 °C.

Blood samples for phenylalanine and tyrosine quantification were placed in heparinized tubes and centrifuged at 2,500 rpm, at +4 °C, for 10 min. An aliquot of supernatant was collected, and transferred to a new tube with 35 % 5-sulfosalicylic acid (10:1 vol/vol), and centrifuged at 8000 rpm, at +4 °C, for 5 min.

Blood and brain samples were allowed to react with the same volume of o-phthaldialdehyde reagent (67.1 mg of o-phthaldialdehyde dissolved in 1.0 ml of methanol plus 50 µl of mercaptoethanol and diluted in 9 ml of borate buffer, 0.4 mM, pH 9.5). After a 2-min reaction time, the sample was transferred to the HPLC system coupled with a fluorescence detector (Waters 474 Model). The excitation and emission wavelengths were set at 330 and 480 nm, respectively. Nova-Pack C18 (3.9 x 150 mm) and Sentry Guard Nova-Pack C18 (3.9 x 20 mm; Waters Assoc.) columns were used. The flow rate was 1.2 ml/min. The mobile phase consisted of 35 % methanol in 0.1 M Na-phosphate buffer, pH 6.5.

### Western bolt analysis

**Fig. 1:**
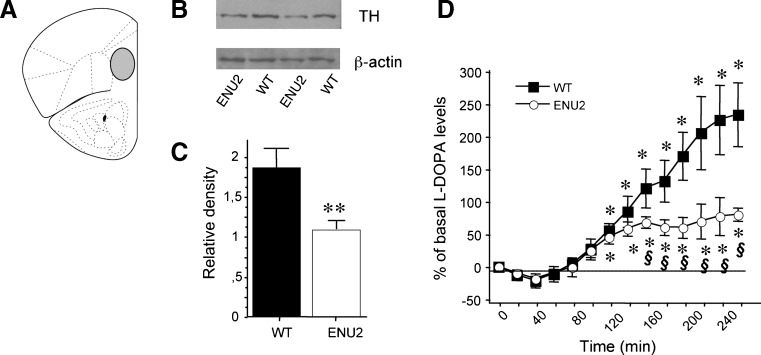
Reduced expression and *in vivo* activity of TH in mpFC of ENU2 mice. (**a**) Schematic representation of mpFC. (**b**) Western blot of TH protein obtained from mpFC of ENU2 and WT mice and (**c**) quantification of protein using chemiluminescence (mean ± S.E.M.) indicated reduced enzyme availability in mutant mice. Detection of β-actin was used as loading control. (**d**) Enzymatic activity of TH was determined measuring accumulation of transient intermediate L-DOPA *in vivo* during continuous infusion of 20 µM NSD-1015. Dialysates were collected at 20-min intervals. Results are expressed as percent changes (means ± S.E.M.) from basal values. Statistical analyses were performed on raw data. Comparison of time course of changes in extracellular levels of L-DOPA in pFC of ENU2 and WT mice reveals reduction of L-DOPA accumulation in ENU2 mice, significant from 160 min onwards. * *P* < 0.05 versus basal values. ***§*** *P* < 0.05 in comparison with WT

Brains of mice from the different groups (WT, n = 8; ENU2, n = 8) were removed, frozen and then fixed vertically on the freeze plate of a freezing microtome maintained at -10 °C. Punches of mpFC (Fig. 1a) were obtained from frozen brain slices as previously reported (Puglisi-Allegra et al 2000) and stored in liquid nitrogen until the day of assay**.** Each mpFC tissue sample was homogenized at 4 °C in lysis buffer [20 mM Tris (pH 7.4), 1 mM EDTA, 1 mM EGTA, 0,1 % Triton X-100] with protease inhibitor cocktail (Sigma-Aldrich, St. Louis, MO, USA). Tissue extract was centrifuged at 12000 g at 4 °C for 15 min. The supernatant fluid was removed and stored at – 80. Samples were heated at 95 °C for 3 min and protein (15 mg) was separated by SDS-PAGE (10 % gel).

Membranes were rinsed in Tris- buffered saline (TBS), then blocked in 5 % non-fat milk in TBS with 0.1 % Tween (TBS-T) for 1.5 h at room temperature in TBS-T and incubated overnight in anti-TH antibody (Chemicon, Temecula, CA, USA) (1:3000 dilution) diluted in 3 % BSA, followed by goat anti-rabbit IgG (H + L) AP conjugate (1:2000 dilution; Santa Cruz Biotechnology, Santa Cruz, CA, USA) in 2 % non-fat milk in TBS-T, and developed with the ECL-R reaction (Amersham). The film signals were digitally scanned and quantified using densitometric image software (imagej 64), normalized for β-actin level.

### In vivo microdialysis

All mice were anesthetized with chloral hydrate, mounted in a stereotaxic frame (David Kopf Instruments, Tujunga, CA) and implanted unilaterally with a guide cannula (stainless steel, shaft outer diameter of 0.38 mm, length 1 mm; Metalant AB, Stockholm, Sweden), fixed with epoxy glue and dental cement, into the mpFC (Fig. 1a; AP, +2.8; L, 0.6; according to the Franklin and Paxinos atlas, 2001). Placement of probes in mpFC was evaluated by methylene blue staining. Only data from mice with correctly placed cannula are reported.

Mice were allowed to recover in their home cage. The probe (2 mm long; MAB 4 cuprophane microdialysis probe, Metelant AB) was introduced 24 h before microdialysis experiments. The mice were lightly anesthetized with chloral hydrate to facilitate manual insertion of the probe into the guide cannula. The dialysis probe was connected to a CMA/100 pump (Carnegie Medicine, Stockholm, Sweden) through PE 20 tubing (Metalant AB) and an ultralow torque dual-channel liquid swivel (model 375/D/22QM; Instech Laboratories, Plymouth Meeting, PA) to allow free movement. Artificial cerebrospinal fluid (147 mM NaCl, 1 mM MgCl, 1.2 mM CaCl_2_, 4 mM KCl) was pumped through the dialysis probe at a constant flow rate of 2 μl/min. The day of the experiments, each animal was transferred to a Plexiglas cylinder provided with microdialysis equipment (Instech Laboratories, Inc.) and with home cage bedding on the floor. Dialysis perfusion was started 1 h later and mice were left undisturbed for 2 h before baseline samples were collected. Dialysate was collected every 20 min. The mean concentration of the three samples collected immediately before treatment (<10 % variation) was taken as basal concentration. Twenty microliters of each dialysate sample were transferred to HPLC systems for analysis.

#### In vivo TH activity

Cortical TH activity in WT (n = 8) and ENU2 (n = 8) mice was determined *in vivo* by the accumulation of L-DOPA in mpFC after inhibition of L-aromatic acid decarboxylase with NSD-1015 (Sigma-Aldrich, St. Louis, MO, USA). Ringer solution containing 20 μM of NSD-1015 was pumped through the dialysis probe at a constant flow rate of 2 μl/min, and dialysates were collected at 20-min intervals for 260 min. L-DOPA was assessed by HPLC coupled to an amperometric detector (Decade II model, Antec Leyden, The Netherlands). The detector potential was set at +700 mV against an Ag/AgCl reference electrode. The mobile phase was previously described (Nakahara et al 2000).

#### L-DOPA treatment

Finally, we evaluated the effect of L-DOPA administration on prefrontal cortical DA response to stress in hyperphenylalaninemic mice. DA, DOPAC (3,4-dihydroxyphenylacetic acid), HVA (homovanilic acid) and NE levels were determined simultaneously, utilizing the HPLC system coupled to a coulometric detector (model 5200 °Coulochem II; ESA, Chelmsford, MA). The conditioning cell was set at +400 mV, electrode 1 at +200 mV, and electrode 2 at -250 mV; the mobile phase was previously described (Pascucci et al 2007). A Nova-Pack C18 column (3.9 x 150 mm; Waters) and a Sentry Guard Nova-Pack C18 pre-column (3.9 x 20 mm) maintained at 30 °C were used. The flow rate was 1.1 ml/min. The detection limit of the catecholamines assay was 0.1 pg.

First, we identified a per se ineffective dose of systematically administered L-DOPA by performing a dose-response study. Naive WT (n = 6) and ENU2 (n = 6) mice were injected i.p. on consecutive days with saline or L-DOPA (0.5, 1, 2.5 mg/kg) and DA and NE in vivo extracellular levels were assessed. Doses were injected in a random order and sufficient time was allowed for neurotransmitter to return to basal levels (no more than 180 min were necessary). No more than two L-DOPA doses were administered daily.

Last, the effect of systemic administration of a per se ineffective dose of L-DOPA on only frontal cortical DA and metabolite response to stress was evaluated, as L-DOPA inability to increase NE frontal cortical extracellular levels. Following collection of baseline samples, animals subjected to the stress experience (WT-sal, n = 8; ENU2-sal, n = 8; ENU2-L-DOPA 0.5, n = 8) were put in a restraint apparatus for 2 h and dialyzate samples were collected every 20 min. The apparatus consisted of an adjustable neck-blocking support mounted on a Plexiglas base and movable U-shaped metal piece that could be fixed to the base at the level of the animal’s hips thus preventing it from turning on its back (Cabib and Puglisi-Allegra 1991).

### Data analysis

The effect of genotype (WT and ENU2) on phenylalanine and tyrosine brain and blood levels and on brain/blood ratios was evaluated by one-way ANOVAs.

Regarding western blot data, the effect of genotype (WT and ENU2) on TH protein levels in mpFC was evaluated by one-way ANOVA.

For microdialysis data, statistical analyses were always carried out on raw data (concentrations: pg/20 µl). Data were presented in figures as percent changes from baseline levels.

The effect of genotype on L-DOPA accumulation in mpFC was analyzed by repeated-measures ANOVA with one between factor (genotype, two levels, WT and ENU2) and one within factor (time, forteen levels, 0, 20, 40, 60, 80, 100, 120, 140, 160, 180, 200, 220, 240, 260 minutes).

The effect of L-DOPA treatment on DA and NE extracellular levels in mpFC of ENU2 and WT mice was analyzed by repeated-measures ANOVAs with one between factor (treatment, four levels, saline, L-DOPA 0.5, 1, 2.5 mg/kg) and one within factor (time, seven levels, 0, 20, 40, 60, 80, 100 and 120 minutes).

The effect of L-DOPA treatment on DA, DOPAC and HVA extracellular levels in mpFC of ENU2 mice subjected to restraint was analyzed by repeated-measures ANOVAs with one between factor (group, three levels, WT-sal, ENU2-sal and ENU2-L-DOPA 0.5) and one within factor (time, seven levels, 0, 20, 40, 60, 80, 100 and 120 minutes).

## Results

### Blood and brain levels and brain/blood ratio of phenylalanine and tyrosine in ENU2 and WT mice

To determine whether high phenylalanine levels inhibit tyrosine transport across the blood-brain barrier, we measured phenylalanine and tyrosine blood and brain levels in ENU2 and WT mice (Table 1). The concentration of phenylalanine was significantly higher in ENU2 than in WT mice, both in blood (~2500 %) and in brains (~1700 %). For tyrosine levels, ENU2 showed reduction of blood and brain levels (~40 %) compared with WT mice. Moreover, phenylalanine blood/brain ratio was significantly reduced, and tyrosine blood/brain ratio was not significantly different, in ENU2 compared with WT mice.

**Table 1:** Blood and brain levels and brain/blood ratios of phenylalanine and tyrosine in WT and ENU2 mice

	WT			ENU2		
	blood	brain	brain/blood ratio	blood	brain	brain/blood ratio
Phenylalanine	92.0 + 4.8	2.2 + 0.2	0.023	2334.1 + 143.7^b^	38.0 + 4.7^b^	0.016 ^a^
Tyrosine	82.8 + 6.1	3.5 + 0.4	0.043	34.2 + 1.7^b^	1.27 + 0.2^b^	0.039

Amino acids levels (μM) in blood and brain samples and blood/brain ratios in WT and ENU2 mice. Values are expressed as means + SEM. ^a^p < 0.05; ^b^p < 0.001 *vs* WT

### Expression and in vivo activity of TH in mpFC of ENU2 and WT mice

Western blot analysis of TH protein (Fig. 1b, c) showed significant difference between the two genotypes (F_1,10_ = 11.25, *p* < .01), revealing a 40 % reduction of TH protein levels in mpFC of ENU2 (1.84 ± .21) in comparison with WT (1.10 ± .06) mice.

Figure 1d reports *in vivo* TH activity in mpFC of ENU2 and WT mice. Statistical analysis revealed significant genotype x time interaction (F_1,182_ = 3.82, p < .05). In WT mice, blockade of aromatic L-amino acid decarboxylase promoted a time-dependent increase of frontal cortical L-DOPA outflow that became significantly higher than basal levels after 120 min, reached maximal levels (233.7 %) at 260 min. ENU2 mice achieved a steady state after 160 min of perfusion, reaching a maximal increase of 80.2 % at 260 min.

### In vivo microdialysis

The two genotypes did not differ for DA cortical basal outflow (WT = 0.95 ± .14 pg/20 ⎧l; ENU2 = 0.98 ± 0.15 pg/20 µl), while NE extracellular levels from mpFC of ENU2 mice were significantly reduced (WT = 2.12 ± .23 pg/20 µl; ENU2 = 1.24 ± 0.19 pg/20 µl; F_1,10_ = 8.64, *p* < .05), as previously reported (Pascucci et al 2009), suggesting that compensatory mechanisms appear to support DA release at the expense of NE in basal conditions.

#### Dose-response curve of L-DOPA

**Fig. 2:**
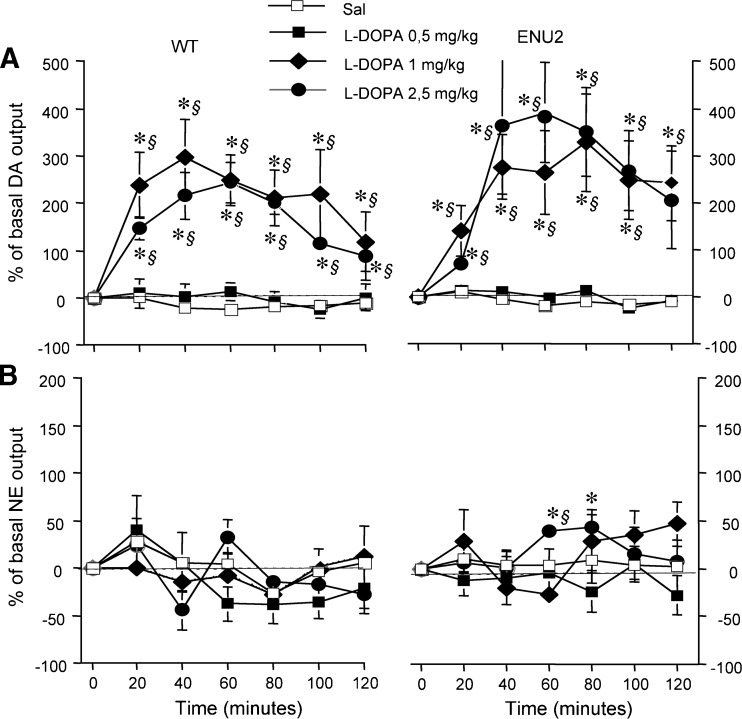
Dose-dependent effect of L-DOPA on frontal cortical catecholamine extracellular levels. Dose-dependent effect of L-DOPA (0.5, 1.0, 2.5 mg/kg i.p.) on DA (**a**) and NE (**b**) outflow in the mpFC of WT and ENU2 mice. Results are expressed as percent changes (means ± S.E.M.) from basal value during 120-min post-injection. Statistical analyses were performed on raw data. Drug was administered to time 0. * *P* < 0.05 vs saline group. ***§*** *P* < 0.05 in comparison with vehicle-injected mice

The dose-dependent effect of L-DOPA treatment on catecholaminergic frontal cortical extracellular release was evaluated (Fig. 2a, b). L-DOPA at a dose of 0.5 mg/kg i.p. had no significant effect on DA outflow in either group of mice, but at the dose of 1 and 2.5 mg/kg i.p. produced significant increase of DA cortical extracellular levels (Fig. 2a).

None of these doses of L-DOPA had an effect on extracellular levels of NE in either group, with the exception of a slight increase at 2.5 mg/kg i.p. in ENU2 mice (Fig. 2b).

#### Effect of L-DOPA treatment on DA cortical neurotransmission in stressed ENU2 mice

**Fig. 3:**
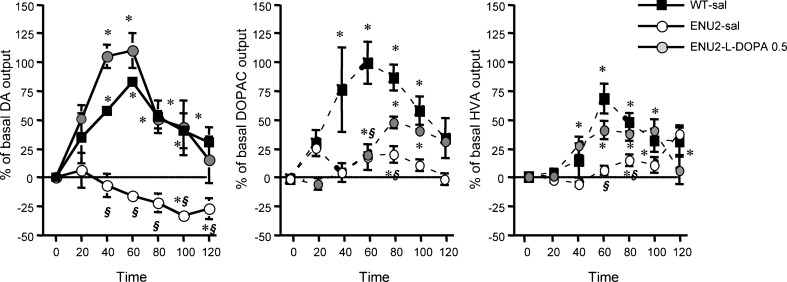
Effect of L-DOPA on dopaminergic cortical neurotransmission in stressed ENU2 mice. Recovery of DA and metabolite response to stress in mpFC of ENU2 mice submitted to 120 min of restraint following systemic administration of 0.5 mg/kg i.p. of L-DOPA. Dialysates were collected at 20-min intervals. Results are expressed as percent changes (means ± SE) from basal values. Statistical analyses were performed on raw data. Drug was administered to time 0. * *P* < 0.05 versus basal values. ***§*** *P* < 0.05 compared with vehicle-injected WT mice

Because L-DOPA treatment was unable to increase NE frontal cortical extracellular levels, effect of per se ineffective dose of L-DOPA was evaluated on frontal cortical DA outflow and turnover (as measured by their major metabolites, DOPAC and HVA) in ENU2 and WT stressed mice (Fig. 3). We compared frontal cortical DA outflow in WT and ENU2 stressed mice following saline or L-DOPA (0.5 mg/kg i.p.) treatment. Statistical analyses revealed a significant group x time interaction (F_18,168_ = 3.15, *p* < .0001). Analyses of DOPAC and HVA extracellular levels in WT, ENU2-sal and ENU2-L-DOPA 0.5 groups revealed significant group x time interactions (DOPAC: F_12,126_ = 3.77, *p* < .0001; HVA: F_12,126_ = 3.90, *p* < .0001).

As previously reported (Pascucci et al 2009), ENU2 mice did not show the typical increase of prefrontal cortical DA outflow. Indeed, DA decreased below basal levels (Fig. 3). Moreover, stress resulted in augmented DOPAC and HVA extracellular levels in WT but not in ENU2 mice (Fig. 3), although DOPAC (WT-sal = 53.34 + 8.87 pg/20 μl; ENU2-sal = 59.20 + 12.61 pg/20 μl; ENU2-L-DOPA 0.5 = 59.88 ± 17.09 pg/20 µl) and HVA (WT-sal = 118,12 ± 21.72 pg/20 µl; ENU2-sal = 154.83 ± 19.24 pg/20 µl; ENU2-L-DOPA 0.5 = 159.65 ± 27.91 pg/20 µl) basal levels did not differ between groups. Nevertheless, in ENU2 mice (similar to WT-sal) treatment with L-DOPA caused an immediate significant increase in DA outflow (20-60 min) followed by a return to basal levels as well as partial but significant increase of DOPAC and HVA extracellular levels.

## Discussion

This study demonstrates that the reduced DA concentrations reported in prefrontal cortical area of PKU mice are mainly due to reduced cortical expression and activity of the TH enzyme, supporting the use of L-DOPA to treat cortical dopaminergic deficits in phenylketonuric subjects.

It is well known that brain amine levels are reduced in PKU patients and mouse models, and DA is the most extensively studied neurotransmitter. In particular, reduced DA availability in mpFC has been proposed as the biochemical mechanism responsible for reduced cognitive performances observed in PKU patients. It has also been proposed that DA cortical deficits stem from decreased levels of amino acid tyrosine (“tyrosine/dopamine” theory), the precursor of DA, and that low levels of tyrosine are a consequence of high phenylalanine levels outcompeting other amino acids for transport across the blood-brain barrier (Diamond et al 1994). Although the “tyrosine/dopamine” theory is strong and empirically supported, controversial data have been reported. Indeed, dietary supplements of tyrosine do not improve cognitive performance in PKU patients (Smith et al 1998), and frontal cortical levels of tyrosine do not return to normal levels when PKU mice are placed on the low phenylalanine diet (Joseph and Dyer 2003). These data indicate that reduced tyrosine availability alone cannot explain the DA cortical deficits, suggesting co-existing of several factors. In this study, we investigated full cortical dopaminergic metabolism in ENU2 mice in order to elucidate phenylalanine-induced interferences at each metabolic step and suggest an easy pharmacological way to raise cortical dopaminergic levels.

Synthesis of catecholamines occurs via hydroxylation of tyrosine to L-DOPA by TH. L-DOPA is rapidly decarboxylated by L-aromatic amino acid decarboxylase to DA, which is then metabolized to NE. Thus, we firstly examined blood and brain availability of DA precursors. Although the presence of high phenylalanine and reduced tyrosine blood and brain levels is well known, the evaluation of brain/blood ratios for tyrosine and phenylalanine in this study is not consistent with the hypothesis of phenylalanine-induced inhibition of amino acid transport to the brain, according to previously reported data (Joseph and Dyer 2003). In fact, we observed a significant reduction of blood and brain tyrosine levels, according to the literature, but found no significant difference in the brain/blood ratio between phenylketonuric and normal mice, suggesting that reduced brain levels of tyrosine reflect low tyrosine blood levels more than reduction of access to the brain. Conversely, when we compared the phenylalanine brain/blood ratio in both groups, we found a trend towards reduced phenylalanine access in the brains of PKU mice, which, however, was unable to prevent high brain phenylalanine levels.

Second, based on demonstrations that tyrosine is not the limiting factor on DA biosynthesis (Joseph and Dyer 2003; Pascucci et al 2009), we investigated cortical availability and activity of TH. Analysis of Western blot data confirmed reduced TH protein levels in mpFC of PKU mice (Joseph and Dyer 2003). Although the decreased protein amount of TH could be an adaptive downregulation in response to reduced dopaminergic synthesis, a faster degradation of the TH protein could not be excluded. Moreover, *in vivo* assay of TH cortical activity (measured as accumulation rate of L-DOPA after blockade of the decarboxylating enzyme) showed significant reduction in the rate of DA synthesis in ENU2 vs WT mice. The reduction of L-DOPA accumulation (66 %) was greater than the 40 % reduction seen in TH protein concentration suggesting other mechanisms causing TH inhibition. This most likely involves a direct inhibitory effect of phenylalanine on mpFC TH activity.

So far our data suggestes three complementary factors are able to explain DA reduced biosynthesis in PKU: decrease of precursor availability to the brain and reduction of protein synthesis and activity of TH enzyme. Recently, we demonstrated that deficits of cortical serotonin biosynthesis in PKU mice are due to phenylalanine -induced inhibition of cortical tryptophan hydroxylase activity (Pascucci et al 2009). These results are in agreement with the hypothesis that phenylalanine influences cortical aminergic transmission by inhibiting activity of enzymes hydroxylating amino acid precursors (Curtius et al 1981; McKean 1972; Ogawa and Ichinose 2006).

Third, based on the reduction of prefrontal cortical TH protein and activity levels in ENU2 mice, we were able to identify L-DOPA, the product of tyrosine hydroxylation, as responsible for increasing DA cortical levels. As previously reported (Pascucci et al 2009), when ENU2 mice were subjected to restraint stress, an environmental challenge known to enhance aminergic release in the mpFC (Page and Lucki 2002; Pascucci et al 2007), they showed deficits in the activation of frontal cortical serotoninergic and dopaminergic transmission and altered noradrenergic responses. In particular, no initial increase of DA release followed by decrease below baseline levels was observed compared with WT, although basal frontal cortical outflow of DA was unaffected. Moreover, DA turnover was also affected by hyperphenylalaninaemia, as shown by reduced DOPAC and HVA extracellular levels in ENU2-stressed mice, although DA basal levels were unaffected.These results suggest compensatory mechanisms are involved that maintain suitable DA metabolism necessary to hold basic physiological functions. However, these mechanisms are unable to sustain the activation solicited by stressful experience.

In order to restore cortical dopaminergic response to stress, we administered L-DOPA, the proximal precursor of DA. The L-DOPA dose-response curves obtained in the ENU2 and WT mice were similar suggesting that the cortical DA metabolic pathway following tyrosine hydroxylation step is intact. Nevertheless, an alternative possibility cannot be excluded: i.e. the L-DOPA-induced DA release depends on serotonergic neurons (Carta et al 2007; Navailles et al 2010; Tanaka et al 1999), wherein L-aromatic amino acid decarboxylase is also present. The same L-DOPA doses were unable to increase frontal cortical release of NE, suggesting an impairment of the conversion of DA to NE in cortical neurons. The administration of a *per se* ineffective dose of L-DOPA (0.5 mg/kg i.p.) affected response to stress in mpFC of ENU2 mice, producing activation of DA and metabolite response. These results show that DA metabolism in the mpFC is very sensitive to L-DOPA treatment, suggesting, under stress challenge, an increase of L-DOPA decarboxylation by DOPA decarboxylase to DA. Although neurological complications related to prolonged treatment with L-DOPA have been reported in Parkinson’s disease patients, the dose used here was well below that associated with abnormal movements in human and in animal models.

Altogether, our data suggests that DA cortical deficits in PKU are due to several factors: reduced precursor cerebral availability, reduced cortical TH protein levels and inhibition of TH cortical activity. Thus, our data raises doubts about using tyrosine in PKU patients. A better approach may be the use of low dose L-DOPA which in the PKU mouse is able to increase cortical DA neurotransmission even in the presence of high blood and brain phenylalanine levels.
